# The C5a anaphylatoxin receptor CD88 is expressed in presynaptic terminals of hippocampal mossy fibres

**DOI:** 10.1186/1742-2094-6-34

**Published:** 2009-11-16

**Authors:** James W Crane, Gilang P Baiquni, Robert KP Sullivan, John D Lee, Pankaj Sah, Stephen M Taylor, Peter G Noakes, Trent M Woodruff

**Affiliations:** 1 Queensland Brain Institute, The University of Queensland, St. Lucia, Brisbane, Qld, 4072 Australia; 2 School of Biomedical Sciences, The University of Queensland, St. Lucia, Brisbane, Qld, 4072 Australia; 3 Centre for Microscopy and Microanalysis, The University of Queensland, St. Lucia, Brisbane, Qld, 4072 Australia

## Abstract

**Background:**

In the periphery, C5a acts through the G-protein coupled receptor CD88 to enhance/maintain inflammatory responses. In the brain, CD88 can be expressed on astrocytes, microglia and neurons. Previous studies have shown that the hippocampal CA3 region displays CD88-immunolabelling, and CD88 mRNA is present within dentate gyrus granule cells. As granule cells send dense axonal projections (mossy fibres) to CA3 pyramidal neurons, CD88 expression could be expressed on mossy fibres. However, the cellular location of CD88 within the hippocampal CA3 region is unknown.

**Methods:**

The expression of CD88 within the hippocampal CA3 region was characterized using dual-immunolabelling of hippocampal sections prepared from Wistar rats. Immunolabelling for CD88, using a monoclonal antibody, was combined with immunolabelling for markers of astrocytes (GFAP), microglia (IBA1), presynaptic proteins (synaptophysin and synapsin-1) and preterminal axons (neurofilament). In addition, electron microscopy was performed on peroxidase-visualized CD88-immunolabelling to determine its cellular localisation within the CA3 region.

**Results:**

Dense CD88-immunolabelling was observed within the *stratum lucidum *of the CA3, consistent with the presence of CD88 on mossy fibres. Labelling for CD88 rarely co-localized with astrocytes or microglia, but was highly co-localized with presynaptic proteins. Electron microscopy revealed CD88-immunolabelling was localized to large presynaptic terminals within the *stratum lucidum*.

**Conclusion:**

These results demonstrate that CD88 is expressed on presynaptic terminals of mossy fibres within the CA3 region of the hippocampus. Although the role of CD88 on mossy fibres remains to be established, their involvement in synaptic/cellular plasticity, and in cognitive disorders such as Alzheimer's disease deserves investigation.

## Background

The complement system is an integral part of the innate immune system activated in response to tissue injury and invading pathogens [1]. Consisting of over thirty fluid-phase and membrane-associated proteins, a major function of the complement systems is cytotoxic destruction of invading pathogens, via the formation of the membrane attack complex [2,3]. However, due to the release of soluble anaphylatoxins such as C3a and C5a, the complement system also initiates and maintains inflammatory responses [1]. For many years the complement system was thought confined to the periphery, it is now recognised that complement factors are expressed in the CNS [2,4,5]. Furthermore, the complement system has been implicated in a number of neurodegenerative disorders, such as Alzheimer's disease, Huntington's disease and amyotrophic lateral sclerosis [2,4,6-10].

The anaphylatoxin C5a, a 74 amino acid glycoprotein, functions primarily as a pro-inflammatory mediator [3,4]. In the periphery, the release of C5a results in a host of inflammatory responses, including increased vascular permeability, chemotaxis of inflammatory cells, and the release of cytokines and chemokines [11]. These responses are primarily mediated via a C5a-selective seven-transmembrane G-protein coupled receptor (termed CD88) [12]. This C5a-receptor is expressed on a wide range of peripheral cells, including neutrophils, monocytes, activated mast cells, endothelial cells, and vascular smooth muscle cells [12]. In the CNS, CD88 can be found on astrocyctes, microglia and neurons in normal human and mouse brains [10,13-19]. Neuronal expression of CD88 has been reported within the *cornus ammonis *sub-fields (CA1 - 3) of the hippocampus, the dentate gyrus, the neocortex, and the cerebellum [18]. Similarly, *in situ *hybridization has demonstrated CD88 mRNA within neurons of the neocortex, cerebellum and dentate gyrus [18].

Granule cells of the dentate gyrus send axonal projections (the mossy fibres) that terminate on CA3 pyramidal neurons within the *stratum lucidum*, a layer lying immediately dorsal to CA3 pyramidal neurons [20]. These strong, excitatory synapses formed by mossy fibres on CA3 pyramidal neurons are critical for the normal function of the hippocampus, and dysfunction of this synapse is thought to contribute to psychiatric disorders such as depression and schizophrenia [20,21]. Although CD88 expression has been reported in the CA3, its precise cellular location has not been fully characterised. The presence of CD88 mRNA within dentate gyrus granule cells [18] suggests CD88 might be expressed on mossy fibres within the CA3 region. Indeed, upon close examination of previous reports [18], CD88 immunolabelling within the human hippocampus does appear to be located within the *stratum lucidum*. Determining whether CD88 is located presynaptically on mossy fibres, or postsynaptically on CA3 pyramidal neurons, is critical to our understanding of its function in this region. Therefore, the present study sought to characterize CD88 expression within the CA3 region of the rat hippocampus with the use of dual-immunolabelling and electron microscopy.

## Methods

### Experimental animals

All results were obtained from male Wistar rats (postnatal days 30-37) housed under standard laboratory conditions with a 12-hour light/dark cycle and food and water available *ad libitum*. All procedures were carried out in accordance with protocols approved by the University of Queensland Animal Ethics Committee.

### Western Blotting

Hippocampal homogenates were separated on a 10% sodium dodecyl sulphate polyacrylamide gel and electro-transferred to a nitrocellulose membrane (Pall). Membranes were blocked for one hour at room temperature in 5% bovine-serum-albumin (BSA) in tris-buffered saline-Tween 20 (TBS-T) prior to incubation with a monoclonal mouse anti- rat CD88 antibody (1:1000; clone R63, Hycult Biotechnology, Netherlands) overnight at 4°C. Membranes were washed (3 × 10 min) in TBS-T before being incubated for one hour at room temperature with goat anti-mouse horseradish peroxidase secondary antibody (1:10000; GE Healthcare, USA). Immunoblots were visualized by ECL chemiluminescence (GE Healthcare).

### Fluorescence Immunohistochemistry

Animals (n = 8) were perfused trans-cardially with 2% sodium nitrite solution (in 0.1 M phosphate-buffer, pH 7.4) followed by 100 ml of 4% formaldehyde (in 0.1 M phosphate-buffer, pH 7.4). All brains were then removed and postfixed overnight in 4% formaldehyde at 4°C, followed by cryoprotection in 20% sucrose (in 0.1 M phosphate-buffered saline, pH 7.4) overnight at 4°C. Some brains (n = 4) were infiltrated in a 1:1 solution of sucrose and cryo-protectant medium (OCT Tissue-Tek, USA) overnight. They were then embedded in 100% OCT, snap frozen in liquid nitrogen and stored at minus 20°C prior to the preparation of coronal sections (16 μm, Leica CM1850 cryostat, Germany). For the remaining brains (n = 2), coronal sections (50 μm) were prepared on a freezing sliding-microtome (Leica SM 2000R, Germany).

All sections were washed in 0.1% phosphate-buffered saline (PBS) and blocked in PBS containing 3% bovine serum albumin (BSA, Sigma-Aldrich, MO, USA). Sections were then incubated in a monoclonal mouse anti-CD88 antibody (1:500 in PBS containing 1% BSA, 4°C; Clone R63, Hycult Biotechnology) for 48 hours, after which they were washed in PBS and incubated overnight at 4°C in a solution of biotinylated donkey anti-mouse antibody (1:300 in PBS containing 1% BSA; Jackson ImmunoResearch, USA). After washing in PBS, sections were incubated in one of the following primary antibodies (made up in PBS containing 1% BSA). Microglia were labelled with a rabbit anti-ionizing calcium-binding molecule antibody (IBA1, 1:600, Biocare Medical, USA). Astrocytes were labelled with a rabbit anti-glial fabrillary acidic protein antibody (GFAP, 1:100; DAKO, Denmark). Presynaptic terminals were labelled using two antibodies directed against protein components of the presynaptic vesicular-release machinery: rabbit anti-synaptophysin antibody (1:100, DAKO), or rabbit anti-synapsin-1 antibody (1:100, Sigma-Aldrich). Preterminal portions of axons were labelled with neurofilament-200 kDa antibody (1:500, Sigma-Aldrich). Following an overnight incubation at 4°C, all sections were washed thoroughly in PBS and incubated in a mixture of Alexa 568-conjugated streptavidin (1:300 in PBS containing 1% BSA, Invitrogen, CA, USA) and Alexa 488-conjugated goat anti-rabbit antibody (1:300 in PBS containing 1% BSA, Invitrogen). Following a wash in PBS, 16 μm thick sections were incubated for 5 min in 4, 6-diamidino-2-phenylindole (DAPI, 1:15000) and then washed with PBS. All sections were cover-slipped in fluorescence mounting medium (DAKO, Denmark), sealed with acrylic and stored at 4°C. Images were acquired using an Axio Imager 319 microscope equipped with a Zeiss AxioCam MRm camera.

### Immunohistochemistry - electron microscopy

Animals (n = 2) were perfused transcardially with 2% sodium nitrite solution (in 0.1 M phosphate buffer, pH 7.4) followed by 50 ml of 4% formaldehyde containing 0.5% glutaraldehyde (in 0.1 M phosphate buffer, pH 7.4). Brains were then removed and postfixed overnight in the same fixative at 4°C and then washed in PBS, after which coronal forebrain (50 μm) sections were cut using a vibratome (Leica VT1000S). All sections were washed (4 × 10 min) in PBS, incubated in a 3% BSA/PBS solution and then incubated in mouse anti-CD88 antibody (1:500 in PBS containing 1% BSA, 4°C; Hycult Biotechnology) for 48 hours. Sections were then washed in PBS and incubated for 2 hours in a solution of biotinylated donkey anti-mouse antibody (1:300 in PBS containing 1% BSA. Jackson ImmunoResearch). After further PBS washes sections were placed into a solution of avidin-biotin-horseradish peroxidase complex (6 μl A and 6 μl B/ml of 1% BSA in PBS; Vector Elite ABC kit, Vector Laboratories, USA) for 2 hours. This was followed by PBS washes (2 × 5 min) and a sodium acetate buffer (pH = 6.0) wash for 5 min. Sections were exposed for 15 min to a 2% NiSO_4 _solution (in sodium acetate buffer) containing 2 mg/ml D-glucose, 0.4 mg/ml NH_4_Cl and 0.025% 3,3-diaminobenzidine (DAB). This was followed by another incubation in the same solution but with the addition of glucose oxidase (0.2 μl/ml). The subsequent production of H_2_O_2 _initiates the peroxidase reaction resulting in the deposition of nickel-DAB. At a suitable time point, based on the background level of staining seen, all sections were washed in sodium acetate buffer to stop the reaction, and then washed thoroughly in PBS (3 × 10 min). Sections were then stained in 1% osmium tetroxide, dehydrated through a graded series of acetone, and processed into epon resin using a microwave (Pelco Biowave). Sections were flat embedded in resin which was polymerized at 60°C for 48 hr. Ultrathin sections (70 nm thick) were cut using an ultra-microtome (Lecia EMUC6) and collected onto grids, stained with uranyl acetate and lead citrate, and examined with a JEOL 1010 electron microscope.

## Results

### CD88 is expressed within the *stratum lucidum *of the hippocampus

**Figure 1:**
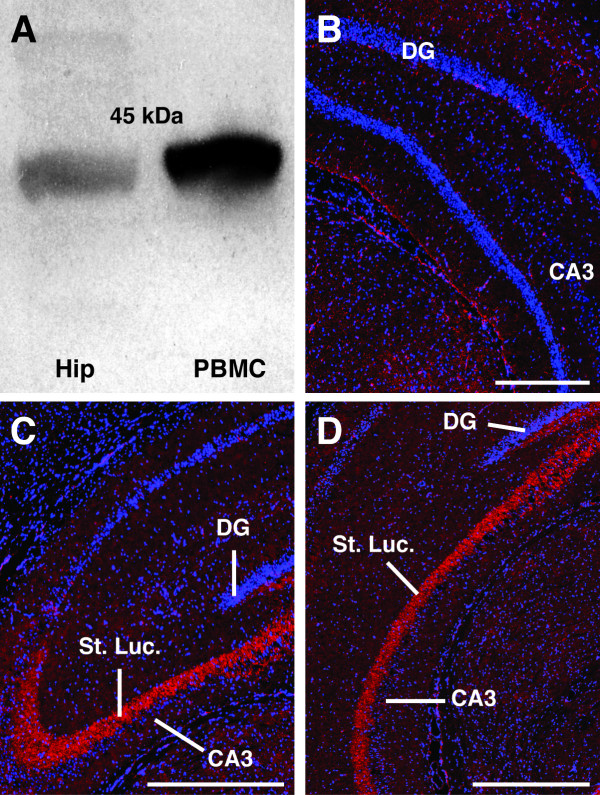
**CD88 expression within the hippocampal *stratum lucidum***. (A) Western blot demonstrating the presence of CD88 protein within the rat hippocampus (Hip). A single band was observed at 45 kDa. A similar immuno-reactive band was found in peripheral blood mononuclear cells (PBMC) that are known to express CD88. (B) Control section immunolabelled without mouse anti-ratCD88 antibody. No labelling was present within the *stratum lucidum *in these sections. (C and D) Intense immunolabelling for CD88 was observed within the *stratum lucidum *(St. Luc.) of the hippocampus (red). This immunolabelling was seen throughout the rostrocaudal extent of the hippocampus: (C) rostral hippocampus, (D) caudal hippocampus. Blue labelling in all sections is DAPI-stained nuclei. CA3, *cornu ammonis *3; DG, dentate gyrus; St. Luc., *stratum lucidum*. Scale bar = 200 μm.

To confirm previous reports that CD88 is expressed within the rat hippocampus [18], western blots were performed. These blots revealed a single band at 45 kDa, the predicted size of glycosylated CD88. A similar band was also observed in lanes containing peripheral blood mononuclear cells; a population of cells known to express cell surface CD88 (Figure 1A).

We next performed immunolabelling for CD88 to determine the location of these receptors within the rat hippocampus. In contrast to sections immunolabelled without the inclusion of the primary antibody (Figure 1B), strong immunolabelling for CD88 was observed within the *stratum lucidum *of the CA3 region. This labelling was seen in every animal and extended across the entire rostrocaudal extent of the CA3 region (Figure 1C and 1D).

### CD88 expression is rarely co-localized with astrocytes and microglia

**Figure 2:**
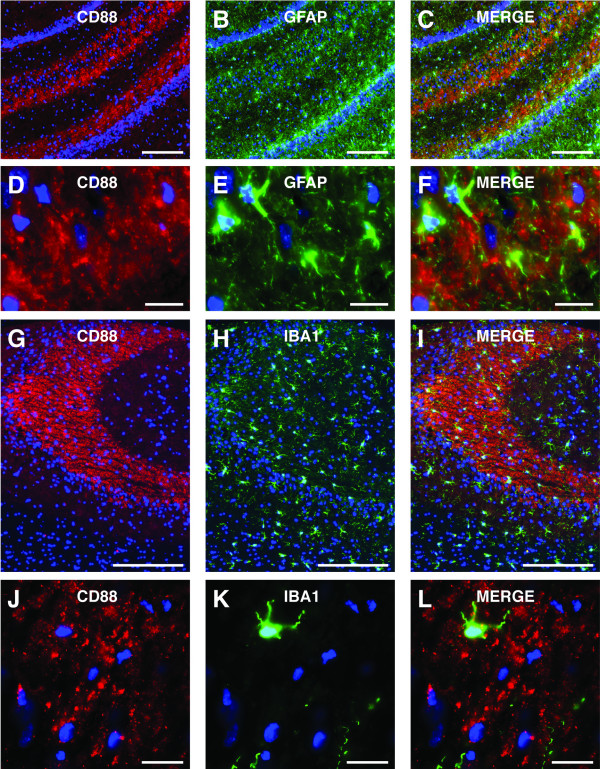
**CD88 in the *stratum lucidum *rarely co-localised with astrocytes and microglia**. (A-F) Immunolabelling for CD88 (red) in the *stratum lucidum *of the hippocampus rarely co-localised with immunolabelling for GFAP (a marker of astrocytes) (green). Although GFAP-positive cells expressed CD88, the vast majority of CD88 immunlabelling in the *stratum lucidum *did not co-localize with GFAP. (G-L) Immunolabelling for CD88 (red) in the *stratum lucidum *of the hippocampus rarely co-localised with immunolabelling for IBA1 (a marker of microglia) (green). Although IBA1-positive cells expressed CD88, the vast majority of CD88 immunolabelling in the *stratum lucidum *did not co-localize with IBA1. GFAP, glial fabrillary acidic protein; IBA-1, ionizing calcium-binding molecule. Scale bars = A-C, 200 μm; D-F, 20 μm; G-I, 200 μm; J-L, 20 μm.

CD88 can be expressed by astrocytes, microglia and neurons [4,5]. To determine whether the CD88 in the *stratum lucidum *was located on astrocytes or microglia we performed dual immunolabelling for CD88 and either GFAP (a marker of astrocytes) or IBA1 (a marker of micoglia) (Figure 2A-L). Astrocytes were found within the *stratum lucidum *(Figure 2B and 2E). However, while astrocytes expressed CD88, the vast majority of CD88 labelling within the *stratum lucidum *was not co-localized with astrocytes (Figure 2C and 2F). Similarly, although microglia were present within the *stratum lucidum *(Figure 2H and 2K) and expressed CD88, the vast majority of CD88 labelling within the *stratum lucidum *was not co-localized with microglia (Figure 2I and 2L).

### CD88 in the *stratum lucidum *co-localizes with presynaptic markers

**Figure 3:**
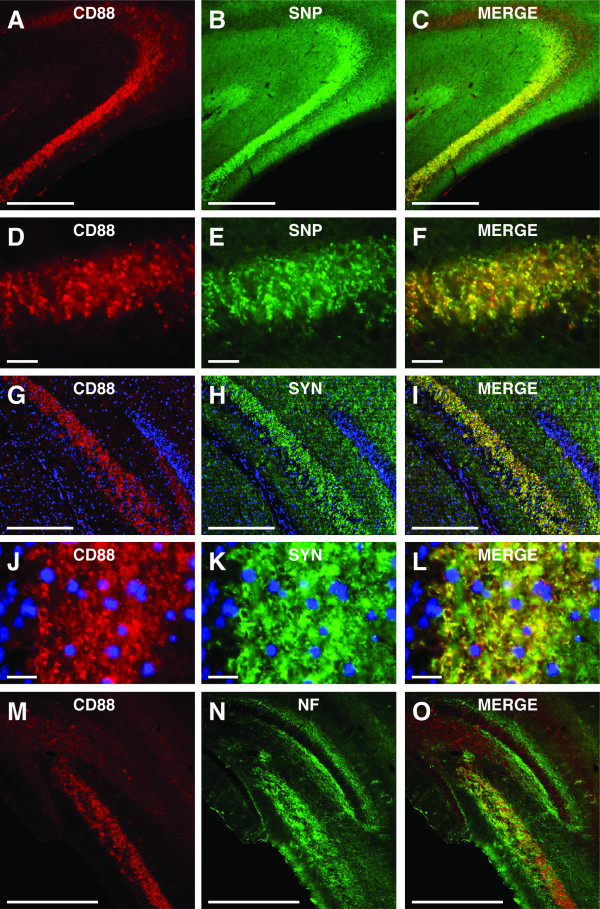
**CD88 in the *stratum lucidum *co-localised with presynaptic proteins**. (A-F) Immunolabelling for CD88 (red) in the *stratum lucidum *displayed almost complete co-localization with the immunolabelling for the presynaptic protein synaptophysin (SNP)(green). There was a highly degree of co-localization of immunolabelling for CD88 and synaptophysin (yellow/orange) within the *stratum lucidum *of the hippocampus. (G-L) Immunolabelling for CD88 (red) in the *stratum lucidum *of the hippocampus co-localized with immunolabelling for the presynaptic protein synapsin-1 (SYN) (green). There was a highly degree of co-localization of immunolabelling for CD88 and SYN (yellow/orange) within the *stratum lucidum *of the hippocampus. (J-O) Immunolabelling for CD88 (red) did not co-localize with immunolabelling for neurofilament (NF, a marker of preterminal portions of the axon) (green). Immunolabelling for NF within the stratum lucidum did not co-localize with immunolabelling for CD88. NF, neurofilament; SNP, synaptophysin; SYN, synapsin. Scale bars = A-C, 200 μm; D-F, 20 μm; G-I, 200 μm; J-L, 20 μm; M-O, 200 μm.

The above results indicate that CD88 within the *stratum lucidum *is located on neuronal membranes. The *stratum lucidum *contains a dense bundle of axons (termed the 'mossy fibres') that originate from the granule cells within the dentate gyrus [20]. These axons form specialized synaptic connections with proximal portions of the apical dendrites of CA3 pyramidal neurons [20]. In the present study, the pattern of CD88 immunolabelling seen within the hippocampus (Figure 1C and 1D) was identical to that seen when mossy fibres are visualised using a Timm's stain [20]. As such, we sought to determine whether CD88 within the *stratum lucidum *was co-localised with two different components of the presynaptic-release machinery: synaptophysin and synapsin-1 (Figure 3A-L).

As expected, there was a dense labelling for synaptophysin within the *stratum lucidum*, consistent with its expression within mossy fibre terminals (Figure 3B and 3E). In contrast to the results seen with GFAP and IBA1, CD88 labelling was almost entirely co-localized with synaptophysin labelling (Figure 3A-F). A similar result was obtained from dual immunolabelling for CD88 and synapsin-1 (Figure 3G-L). Dense labelling for synapsin-1 was observed within the *stratum lucidum *(Figure 3H and 3K), and this displayed a high degree of co-localization with immunolabelling for CD88 (Figure 3G-L).

To further characterize the presynaptic location of CD88, we co-immunolabelled sections for CD88 and neurofilament-200 kDA (a marker of pre-terminal portions of axons) [22] (Figure 3M-O). There was dense labelling for neurofilament within the *stratum lucidum *(Figure 3N and 3O), consistent with the large bundle of axons present within this region. However, the vast majority of labelling for CD88 did not co-localize with neurofilament (Figure 3M-O).

### Electron microscopy: CD88 is localized to presynaptic terminals

**Figure 4:**
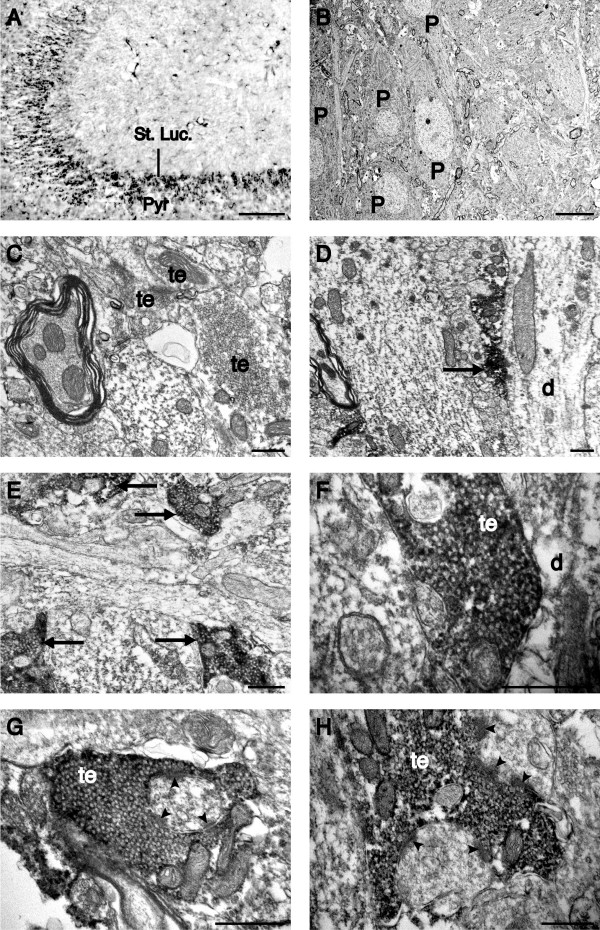
**CD88 is located on presynaptic terminals of the *stratum lucidum***. A) Low power photomicrograph of nickel-DAB visualized CD88 immunolabelling in the *stratum lucidum *(St. Luc.) of the rat hippocampus. Ultrathin sections from labelled *stratum lucidum *and the underlying pyramidal cell layer (Pyr) were further processed for electron microscopy. B) Electron micrograph taken through the pyramidal cell layer of the CA3 region. No nickel deposits were seen on pyramidal cells (P). C) Electron micrograph taken from unlabelled ultrathin sections from the *stratum lucidum*. Note the presence of unlabelled, large presynaptic terminals (te) in the *stratum lucidum*. D-H) Electron micrographs demonstrating nickel-DAB intensified labelling for CD88 localized to presynaptic terminals of the rat *stratum lucidum*. D-F) Presynaptic terminals immunolabelled for CD88 (arrows) were located in close apposition to a dendritic process (d). G-H) Higher magnification electron micrographs of CD88 immunolabelled presynaptic terminals demonstrating their close apposition to postsynaptic densities (arrow head). Scale bars = A, 100 μm; B, 10 μm; C-H, 500 nm.

To further examine the subcellular localisation of CD88 within the *stratum lucidum *we examined nickel-DAB intensified CD88 immunolabelling using electron microscopy (Figure 4A-H). Using light microscopy, immunolabelling for CD88 was seen as punctate nickel-DAB deposits within the *stratum lucidum *(Figure 4A), consistent with the labelling seen with immunoflourescent immunohistochemistry (Figure 1C and 1D). Examination of a adjacent sections using electron microscopy confirmed a lack of CD88 immunolabelling within the pyramidal cell layer of the CA3 region (Figure 4B). Similarly, nickel-DAB deposits were absent from *stratum lucidum *sections that were not immunolabelled for CD88 (Figure 4C). In contrast, in sections immunolabelled for CD88, electron-dense nickel-DAB deposits were localised to large presynaptic terminals in the *stratum lucidum *(Figure 4D-H). These terminals were densely packed with presynaptic vesicles and were present in apposition to dendritic processes (Figure 4D-F). Terminals immunolabelled for CD88 were also seen opposite postsynaptic densities, a marker of glutamatergic synapses (Figure 4G and 4H).

## Discussion

The results presented here clearly demonstrate that the G-protein coupled C5a-receptor (CD88) is located on presynaptic terminals of granule cell axons (mossy fibres) located in the *stratum lucidum *of the CA3 region of the rat hippocampus. Immunolabelling for CD88 revealed a pattern of expression identical to that observed when mossy fibres are visualized with a Timm's stain [20]. Consistent with this, immunolabelling for CD88 almost entirely co-localized with immunolabelling for the presynaptic proteins synaptophysin and synapsin-1. However, CD88 immunolabelling rarely co-localized with neurofilament (a marker of pre-terminal portions of the axon). The localisation of CD88 was further determined using electron microscopy, demonstrating CD88 immunolabelling within large presynaptic terminals of the *stratum lucidum*.

The production and specificity of the CD88 antibody used in this study (monoclonal mouse anti-ratCD88, clone R63) has previously been fully described [23]. Briefly, this antibody was raised against membrane-expressed CD88 in a rat basophilic leukemia cell line (RBL-2H3). The specificity of the antibody raised was the established using flow cytometry and ELISA procedures, with the antibody found to target the N-terminal region (the putative first extracellular domain) of CD88. In the present study, western blot analysis revealed that this CD88 antibody detected a single 45 kDa band from both isolated hippocampus tissue and peripheral blood mononuclear cells. Recently, a second C5a binding receptor has been identified termed C5a-like receptor 2 (C5L2) [24]. This receptor does not appear to couple to G-proteins and is suggested to be either a C5a decoy receptor [25,26] or a functional C5a signalling receptor [27,28]. The C5L2 receptor and CD88 display ~35% homology in their amino acid sequences, and a considerable amount of this homology is located in their N-terminal regions [12]. As the antibody used in the present study binds in the N-terminal region of CD88 we considered the possibility that this antibody also recognized C5L2. To test this we immunolabelled rat hippocampal sections with a specific anti-ratC5L2 antibody [29]. This revealed C5L2 expression on astrocytes, but, consistent with previous reports [29], no C5L2 immunolabelling was observed on mossy fibres of the *stratum lucidum *(data not shown). As such, the immunolabelling seen within the *stratum lucidum *of the rat hippocampus in the present study is due to the presence CD88.

Immunolabelling for CD88 within the *stratum lucidum *of the CA3 region of the hippocampus was found to display a high degree of co-localisation with immunolabelling for the presynaptic proteins synaptophysin and synapsin-1. Both synaptophysin and synapsin-1 proteins are located on synaptic vesicles and are involved in exocytosis of neurotransmitter into the synaptic cleft [30,31]. As such, the co-localization of CD88 immunolabelling with synaptophysin and synapsin-1 immunolabelling indicates that CD88 is located in close proximity to the presynaptic terminals of mossy fibres within the *stratum lucidum*. In contrast, immunolabelling for CD88 was not co-localized with neurofilament immunolabelling. As neurofilaments are almost entirely confined to the preterminal region of axons [22], this result indicates that CD88 expression is confined to the presynaptic terminals of the mossy fibres. This was subsequently confirmed using electron microscopy, in that CD88 immunolabelling was confined to large presynaptic terminals present within the *stratum lucidum*.

The role of CD88 on mossy fibre terminals within the CA3 region of the hippocampus is not known. However, a range of functions for CD88 within the CNS have been proposed, including apoptosis, cytoskeletal plasticity and axodendritic outgrowth, the migration of neural stem cells, and the release of neurotrophins and cytokines [5]. Neuroblastoma cells respond to C5a with an increase in calcium influx [32,33], increased expression of the immediate early gene c-*fos *[32,33], activation of protein kinase-C, and the nuclear translocation of nuclear factor kappa-light-chain-enhancer of activated B cells (NF-κB) [18]. Activation of CD88 is mitogenic for undifferentiated neuroblastoma cells, protects terminally differentiated neuroblastoma cells against amyloid β peptide mediated toxicity [18], and leads to calcium influx and apoptosis in cortical neuronal cultures [14]. However, CD88 activation also appears to protect against glutamate-mediated cell death, possibly via the activation of the mitogen-activated protein kinase cascade [34] and to have an anti-apoptotic function in cultured cerebellar granule cells [13].

Primary neuronal cultures from the hippocampus respond to CD88 activation with an increased calcium influx [18]. Similarly, calcium influx into hippocampal neurons is seen in brain slices treated with a CD88 agonist [14]. Administration of C5a reduces kainic acid-induced excitotoxicity in CA3 pyramidal neurons [35], and CA3 pyramidal neurons of CD88 knockout mice are more susceptible to excitotoxicity [36]. Interestingly, C5a has also been suggested to act presynaptically to increase noradrenaline release, a mechanism that might underlie the increased eating seen in response to intra-hypothalamic infusion of C5a [37,38]. Further, systemic administration of a CD88 antagonist has been reported to decrease the pathology, increase synaptophysin immunolabelling in the CA3 *stratum lucidum *and enhance behavioural outcomes in mouse models of Alzheimer's disease [6]. These previous findings provide evidence that C5a plays a neuromodulatory role in the CNS. That C5a also acts through CD88 to modulate neurotransmission between mossy fibres and CA3 pyramidal neurons is, therefore, an intriguing possibility.

The generation of new neurons is thought to be a significant form of plasticity within the CNS. Recently, cultured neural stem cells have been shown to express CD88. This expression was also found on doublecortin-positive cells within the sub-ventricular zone (the primary site of neurogenesis in the rodent brain) and rostral migratory stream [39]. The subgranular zone of the hippocampal dentate gyrus is a site of neurogenesis in the adult mammalian brain. Incorporation of these neurons into the dentate gyrus and their subsequent synaptic connection to CA3 pyramidal neurons appears to be involved in some forms of hippocampal-dependent learning [21,40]. Given that C5a acts as a strong chemotactic signal in the periphery [1,11], it is possible that it also acts as a guidance cue for the axons of newly formed granule cells.

## Conclusion

The results of the present study clearly demonstrate that CD88 expression within the CA3 region of the rat hippocampus is primarily located within the *stratum lucidum *of the rat hippocampus. Immunolabelling for CD88 co-localized with immunolabelling for the presynaptic proteins synaptophysin and synapsin-1, suggesting that CD88 is located on terminals of mossy fibre axons originating from dentate gyrus granule cells. This result was confirmed using electron microscopy, demonstrating that CD88 immunolabelling was confined to large presynaptic terminals of the *stratum lucidum*. As such, the C5a-receptor CD88 is well positioned to influence hippocampal function in both diseased and non-diseased states.

## Competing interests

The authors declare that they have no competing interests.

## Authors' contributions

JWC and TMW jointly conceived the project in discussions with SMT, and performed preliminary experiments in the laboratory of SMT. GB and JDL preformed immunolabelling and Western blots in the laboratory of PGN, under his and TMW supervision. JWC and RS performed additional immunolabelling in the laboratory of PS. JWC prepared the manuscript with assistance of TMW, PGN, SMT, JDL and PS. All authors have read and approved the final version of this manuscript.
